# Changes Induced by Aging and Long-Term Exercise and/or DHA Supplementation in Muscle of Obese Female Mice

**DOI:** 10.3390/nu14204240

**Published:** 2022-10-12

**Authors:** Alejandro Martínez-Gayo, Elisa Félix-Soriano, Neira Sáinz, Pedro González-Muniesa, María J. Moreno-Aliaga

**Affiliations:** 1 Department of Nutrition, Food Science and Physiology, Faculty of Pharmacy and Nutrition, University of Navarra, 31008 Pamplona, Spain; 2 Center for Nutrition Research, University of Navarra, 31008 Pamplona, Spain; 3 CIBER Physiopathology of Obesity and Nutrition (CIBERobn), Carlos III Health Institute (ISCIII), 28029 Madrid, Spain; 4 IdISNA–Navarra Institute for Health Research, 31008 Pamplona, Spain

**Keywords:** myokines, DHA, exercise, aging, inflammation, muscle, inflammaging

## Abstract

Obesity and aging promote chronic low-grade systemic inflammation. The aim of the study was to analyze the effects of long-term physical exercise and/or omega-3 fatty acid Docosahexaenoic acid (DHA) supplementation on genes or proteins related to muscle metabolism, inflammation, muscle damage/regeneration and myokine expression in aged and obese mice. Two-month-old C57BL/6J female mice received a control or a high-fat diet for 4 months. Then, the diet-induced obese (DIO) mice were distributed into four groups: DIO, DIO + DHA, DIO + EX (treadmill training) and DIO + DHA + EX up to 18 months. Mice fed a control diet were sacrificed at 2, 6 and 18 months. Aging increased the mRNA expression of *Tnf-α* and decreased the expression of genes related to glucose uptake (*Glut1*, *Glut4*), muscle atrophy (*Murf1*, *Atrogin-1*, *Cas-9*) and myokines (*Metrnl*, *Il-6*). In aged DIO mice, exercise restored several of these changes. It increased the expression of genes related to glucose uptake (*Glut1*, *Glut4*), fatty acid oxidation (*Cpt1b*, *Acox*), myokine expression (*Fndc5*, *Il-6*) and protein turnover, decreased *Tnf-α* expression and increased p-AKT/AKT ratio. No additional effects were observed when combining exercise and DHA. These data suggest the effectiveness of long-term training to prevent the deleterious effects of aging and obesity on muscle dysfunction.

## 1. Introduction

The world population is becoming older and more obese and as a consequence the prevalence of chronic non-communicable diseases, such as cardiovascular disease, cancer and diabetes is also increasing, being the leading causes of death in the world [1]. Obesity is considered a pandemic worldwide. According to the World Health Organization, 39% of the population was overweight and 13% was obese in 2016 [2] and 1 billion adults are expected to be obese by 2025 [3]. In parallel, life expectancy has increased significantly worldwide over the last century. Several developed countries have life expectancies above 80 years old [4] and the number of people over 80 is expected to triple from 2019 to 2050 [5]. Albeit undoubtedly positive, the increase in life expectancy is not without complications: aging promotes chronic inflammation and is associated with the loss of muscle mass and with body fat accumulation, mainly visceral [6].

Inflammation is a physiological process that has the role of resolving the issue or damage and returning the body to homeostasis [7]. Nonetheless, when inflammation is not resolved, low-grade systemic inflammation becomes a chronic condition, causing systemic damage and altering the function of the organs. Both obesity and aging promote chronic low-grade systemic inflammation, which can increase the risk of developing metabolic disturbances [8,9] and may be one of the main risk factors for the increase in several non-communicable diseases [10].

Skeletal muscle is a plastic and a highly dynamic organ with an important role in regard to inflammation. Two decades ago [11], it was discovered that it can secrete bioactive molecules during exercise, called myokines [12,13]. These molecules are considered one of the main drivers of the health benefits observed with physical exercise [12], such as counteracting the effects of the pro-inflammatory cytokines produced by dysfunctional white adipose tissue (WAT) in obesity [14], or inducing the change of WAT to a more thermogenic (beige) phenotype [15,16]. However, several situations can negatively impact this tissue. A significant amount of skeletal muscle mass and strength are lost with aging (sarcopenia) [17] and obesity can potentiate this process [18]. Therefore, both situations can increase the rate of muscle loss and deterioration, altering myokine production, promoting low-grade systemic inflammation and affecting overall health and life quality. Notably, due to estrogen deficiency, sarcopenia and obesity seem to be accelerated in postmenopausal women [19].

While a relevant number of studies have analyzed the effects of aging and obesity on sarcopenia in humans [20,21,22,23,24], the studies analyzing the mechanisms involved in the alterations induced by the combined effects of aging in an obesogenic environment and potential therapies on the expression levels of genes/proteins related to muscle metabolism and function in mice models are scarce [18]. Therefore, the search for effective strategies and the understanding of the mechanisms involved to improve or restore skeletal muscle mass and/or function and promoting a healthy aging are crucial.

Physical exercise and omega-3 fatty acids have been studied extensively as potential treatments for preventing or reverting chronic inflammation [25,26]. It has been observed that exercise can reduce adiposity and several markers of inflammation [27] and improve skeletal muscle mass and function in healthy elderly people [28], while omega-3 polyunsaturated fatty acids (*n*-3 PUFA) have been shown to have several health benefits, especially in relation to their anti-inflammatory properties [29,30]. Nevertheless, few studies to date have addressed the potential beneficial effects of long-term consumption of *n*-3 PUFA coupled with physical exercise in the context of aging and obesity [31,32,33,34]. In addition, most of the studies analyzing the effects of *n*-3 PUFA or exercise on sarcopenia have been conducted in male mice [35,36,37]. In this respect, we have recently reported that a 16-week resistance training (RT) program (alone or in combination with Docosahexaenoic acid (DHA)) improved the lower and upper limb muscle strength and quality in overweight/obese postmenopausal women [38]. Moreover, recent studies of our group in aged and obese mice have shown that DHA and/or treadmill training can delay the progression of non-alcoholic fatty liver disease and inflammation [39] and that long-term DHA supplementation was able to reduce inflammation in the dysfunctional brown adipose tissue (BAT), as well as the alteration in lipid metabolism biomarkers (total and LDL-cholesterol) that characterizes aging and obesity in mice [40].

Therefore, the aim of the present study was to investigate the effects of aging on the expression of genes and some proteins related to inflammation, muscle damage and regeneration, muscle glucose uptake, fatty acid oxidation and myokine expression, in skeletal muscle of female mice. Moreover, we aimed to determine the potential beneficial effects of long-term dietary supplementation with DHA, regular physical exercise and a combination of both, on the aforementioned genes and proteins, in diet-induced obese and aged female mice.

## 2. Materials and Methods

### 2.1. Animals and Treatments

Seven-week-old female C57BL/6J mice were purchased from Harlan Laboratories (Barcelona, Spain). The animals were housed at the animal facilities of the University of Navarra under controlled conditions (22 ± 2 °C, with a 12 h light–dark cycle, relative humidity, 55 ± 10%). All experiments were performed according to National animal care guidelines and with the approval of the Ethics Committee for Animal Experimentation of the University of Navarra (Protocol 113–15), in accordance with the EU Directive 2010/63/EU. After acclimation, a group of animals (*n* = 26) was fed a normal control diet containing as energy: 20% proteins, 67% carbohydrates and 13% lipids (2014 diet, Harlan Teklad Global Diets, Harlan Laboratories, Indianapolis, IN, USA). These mice were grown and sacrificed at different ages: 2-month-old (*n* = 10), 6-month-old (*n* = 7) and 18-month-old (*n* = 9). Another group of mice was fed ad libitum a high saturated fat diet (HFD) (*n* = 33) containing as energy: 20% proteins, 35% carbohydrates and 45% lipids (5.5% soybean, 39.5% lard; Research Diets Inc., New Brunswick, IN, USA). Animals were fed the HFD for 4 months to induce obesity (from 2 to 6 months old). Next, mice were divided into 4 experimental groups: (1) Obese (DIO) group (*n* = 10) that continued with the HFD up to 18 months; (2) Obese + DHA-rich *n*-3 PUFA (DIO + DHA) group (*n* = 6), fed with the HFD supplemented with a DHA-rich fish oil concentrate, replacing 15% wt/wt of dietary lipids (Research Diets Inc., New Brunswick, USA), up to 18 months; (3) Obese + Exercise (DIO + EX) group (*n* = 8), fed the same HFD as the obese group in combination with a regular treadmill exercise training, up to 18 months; and (4) Obese + DHA-rich *n*-3 PUFA + Exercise (DIO + DHA + EX) group (*n* = 9), fed the HFD supplemented with DHA-rich fish oil concentrate, in combination with a regular aerobic physical exercise training (treadmill exercise), up to 18 months. The DHA-rich *n*-3 PUFA concentrate (SOLUTEX0063TG, Solutex, Spain) contained 683.4 mg DHA/g and 46.7 mg EPA/g, with a total content of *n*-3 PUFA of 838.9 mg/g as TG. Given that the DHA-rich *n*-3 PUFA concentrate contained mixed tocopherols (2 mg/g of Covi-ox^®^ T-79EU) to prevent oxidation, the HFD of the DIO and DIO + EX groups was supplemented with the same amount of tocopherol mix. The different HFDs (prepared by Research Diets, Inc., New Brunswick, NJ, USA) were vacuum-sealed in 2.5 kg plastic bags and kept frozen (−20 °C) until used to avoid rancidity. The detailed composition of the diets can be found in the Supplementary Materials of a previous article of our group [39].

Before the sacrifice, whole-animal lean mass was measured by magnetic resonance (EchoMRI-100-700; Echo Medical Systems, Houston, TX, USA), as previously described [41]. Mice were sacrificed with an average of 30 hours after the last bout of training and after overnight fasting and gastrocnemius and soleus muscles were collected, weighed and placed in liquid nitrogen before being stored at −80 °C.

### 2.2. Training Protocol

The DIO + EX and DIO + DHA + EX groups were subjected to a treadmill training program (LE8710M, Panlab, Barcelona, Spain) from 6 until 18-month-old. Before the beginning of the treadmill training, mice were allowed to adapt to the treadmill by running for 10 min on 2 consecutive days (first day at 3 m/min; second day at 4.8 m/min). From months 6 to 10, mice were subjected to the following training program (3 m/min for 5 min, increased to 4.8 m/min for 5 min and then reaching a maximum of 7.2 m/min for 20 min at 0% slope) for 3 alternate days/week. At 10 months of age, the number of sessions and the speed of the training were increased to 5 days per week during 5 weeks with the following protocol: speed and running time were started at 5 m/min for 5 min, increased to 8 m/min for 5 min and then reached a maximum of 12 m/min for 20 min at 0% slope. During the next 7 months, the exercise protocol was maintained, but the number of sessions was reduced from 5 to 3 days a week. The mice in the non-exercise groups were left on the treadmill, without running, for the same period of time as the exercise groups. The exercise bouts started during the last hours of their light cycle (late light/rest phase), in order to cause a minimum impact on the rest phase of the mice.

### 2.3. mRNA Expression (Real-Time PCR)

Total RNA was extracted from mice gastrocnemius muscle using TRIzol^®^ reagent (Invitrogen, CA, USA) according to the manufacturer’s instructions and then reverse transcribed to cDNA as previously described [42]. RNA concentrations and quality were measured by Nanodrop Spectrophotometer 1000 (Nanodrop Technologies, Inc. Wilmington, NC, USA). RNA was then incubated with DNase I (RapidOut DNA Removal kit, Thermo Fisher Scientific, Waltham, MA, USA) for 30 min at 37 °C and reverse transcribed to cDNA using the High-Capacity cDNA Reverse Transcription Kit (Applied Biosystems, Thermo Fisher Scientific) according to the manufacturer’s instructions. Real-time PCR was performed using the Touch Real-Time PCR System (CFX384, BIO-RAD, Hercules, CA, USA). Tumor necrosis factor alpha (*Tnf-α*), Interleukin 6 (*Il-6*), Interleukin 10 (*Il-10*), Caspase-3 (*Cas3*), Caspase-8 (*Cas8*), Caspase-9 (*Cas9*), Muscle-specific RING finger protein 1 (*Murf1/Trim63*), Muscle atrophy F-Box (*Atrogin-1/Mafbx*), Myogenic factor 5 (*Myf5*), Myogenic differentiation 1 (*Myod*/*Myod1*), Myogenin/Myogenic factor 4 (*Myog*), Glucose transporter 1 (*Slc2a1/Glut1*), Glucose transporter 4 (*Slc2a4/Glut4*), Acetyl-CoA Oxidase 1 (*Acox*), Carnitine palmitoyltransferase 1B (*Cpt1b*), Myostatin (*Mstn*), Meteorin-like *(Metrnl*), *Adiponectin* (*Adipoq*) and Fibronectin type III domain containing 5 (*Fndc5*) were analyzed using Power SYBR Green PCR (Bio-Rad, München, Germany). Ribosomal Protein Lateral Stalk Subunit P0 (*Rplp0/36b4*) was used as housekeeping gene. Relative expression of the specific genes was determined using the 2^−ΔΔCt^ method [43]. Sequences of the mouse forward and reverse primers are shown in Table 1.

### 2.4. Protein Extraction and Western Blot Analysis

Gastrocnemius muscle samples were homogenized with lysis buffer (8 mmol/L NaH_2_PO_4_, 42 mmol/L Na_2_HPO_4_, 1% sodium dodecyl sulfate (SDS), 0.1 mol/L NaCl, 0.1% NP_40_, 1 mmol/L NaF, 10 mmol/L sodium orthovanadate, 2 mmol/L phenyl-methyl-sulphonylfluoride (PMSF), 10 mM ethylenediaminetetraacetic acid (EDTA) and 1% protease inhibitor cocktail 1 (MilliporeSigma, Darmstadt, Germany)) and centrifuged at 13,000 rpm for 15 min to obtain the supernatant fraction containing the proteins. Protein extracts were quantified with the BCA protein assay kit (Thermo Fisher Scientific, Waltham, MA, USA) to determine their concentration. Protein extracts (25 µg) were separated by electrophoresis on 10% sodium dodecyl sulfate–polyacrylamide gel. Then, proteins were electroblotted from the gel to nitrocellulose membranes (Amersham^TM^ Protran^TM^, GE Healthcare Life Science). Efficient protein transfer was monitored by Ponceau S stain. Next, membranes were blocked (5% bovine serum album) for 1 h at room temperature and incubated overnight at 4 °C with specific primary antibodies at 1:1000 against: Phospho-AKT (p-AKT) (rabbit, Ser473), AKT (rabbit), Phospho-Acetyl-CoA Carboxylase (p-ACC) (rabbit, Ser79), Acetyl-CoA Carboxylase (ACC) (rabbit) (Cell Signaling Technology, Danvers, MA, USA) and β-Actin (mouse, Sigma-Aldrich). After the incubation, corresponding peroxidase conjugated secondary antibodies (anti-rabbit and anti-mouse, Cell Signaling Technology, Danvers, MA, USA) were used at 1:5000 for 1 h at room temperature. Finally, proteins were detected using the C-DiGit chemiluminescent digital scanner and quantitated by densitometry analysis using the Image Studio Lite software (both from LI-COR Biosciences, Lincoln, NE, USA). The results are expressed in relation to the corresponding control value, which was set to 1.

### 2.5. Statistical Analysis

Statistical analyses were performed using GraphPad Prism version 8.00 for Windows (GraphPad Software, La Jolla, CA, USA) and STATA 17 (Stata, College Station, TX, USA). All the results are expressed as mean (SD). Normality of residuals was studied with the Shapiro-Wilk test. Depending on the normality, the effects of aging on lean mass, protein expression and gene expression at 2, 6 and 18 months were analyzed by either one-way ANOVA followed by Tukey’s multiple comparisons test or by Kruskal-Wallis test followed by Dunn’s multiple comparisons test. Due to the factorial design of the study, the effects of DHA supplementation and treadmill training on 18-month-old DIO mice were analyzed by either two-way ANOVA or by permutation test [44], depending on the normality. If a significant interaction was found between the two main factors (Exercise x DHA), either contrast analyses or Mann-Whitney U tests were performed depending on the normality, in order to differentiate the group effects. With the aim of measuring the effect size of aging and of the treatments, Cohen’s d were calculated. Statistical significance was set at *p* < 0.05.

## 3. Results

### 3.1. Effects of Aging on Skeletal Muscle

#### 3.1.1. Effects on Whole Body Lean Mass and Muscle Relative Weights

Body weight (BW) increased significantly with age, as described in a previous study of our group [45]. Absolute lean mass also increased significantly from young (2-month-old) to adult (6-month-old) mice (*p* < 0.001, d = 3.48) and from adult to old (18-month-old) mice (*p* = 0.036, d = 1.29). When lean mass was expressed as a percentage of BW, old mice had 25.8% and 24.5% less lean mass than young and adult mice (*p* < 0.001), respectively. As reported in a previous study of our group [45], the absolute weight of the gastrocnemius muscle increased with age, while the relative weight tended to decrease (not significant). Although the absolute weight of the soleus muscle did not change with age (data not shown), the relative weight of this muscle was significantly lower in adult (*p* = 0.030, d = −1.35) and old mice (*p* = 0.007, d = −1.72) compared with young mice (Table 2). When comparing the old lean mice to old obese (DIO) mice, the DIO mice had significantly more lean mass (*p* < 0.001) but less relative lean mass (*p* < 0.001) and less relative weight of the gastrocnemius and soleus muscle (*p* < 0.001) (Figure S1A,B).

#### 3.1.2. Effects on mRNA Expression of Genes Related to Inflammation, Muscle Damage and Muscle Regeneration

**Figure 1:**
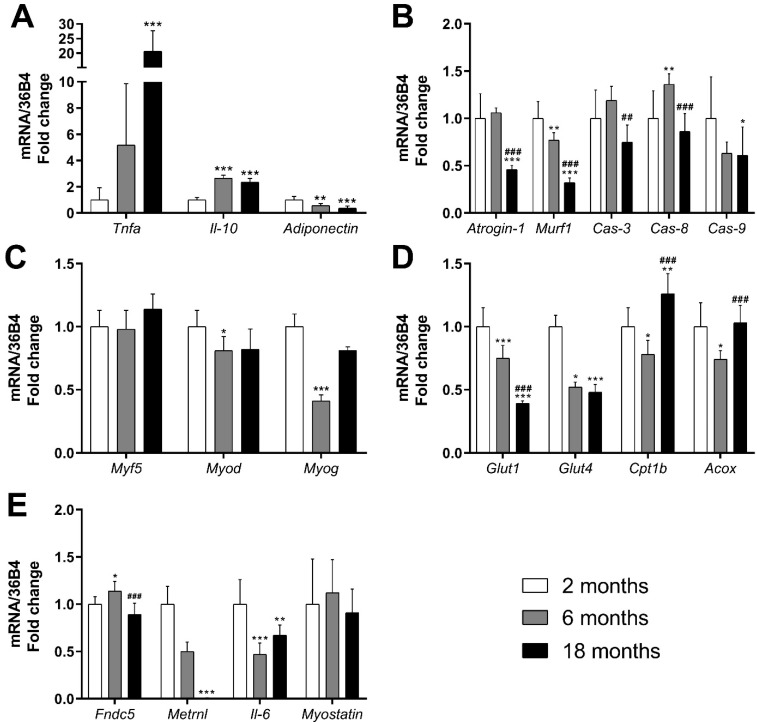
Effects of aging (2, 6 and 18 months) on the mRNA expression of genes related to inflammation (**A**), muscle damage (**B**), muscle regeneration (**C**), glucose uptake and fatty acid oxidation (**D**) and myokine expression (**E**) on the gastrocnemius muscle of C57BL/6J female mice fed a standard diet. Data presented as mean (SD); *n* = 7–10. * *p* < 0.05, ** *p* < 0.01, *** *p* < 0.001 vs. 2-month-old mice; ^##^ *p* < 0.01, ^###^ *p* < 0.001 vs. 6-month-old mice.

TNF-α is a highly pro-inflammatory cytokine associated with several pathological conditions [46]. As shown in Figure 1A, significant increases in *Tnf-α* mRNA were detected in the gastrocnemius muscle of old mice compared with young ones (*p* < 0.001, d = 4.10).

On the contrary, *IL-10* is a cytokine with important anti-inflammatory properties [47]. Interestingly, *Il-10* mRNA expression increased in both aged (*p* < 0.001, d = 5.58) and adult mice (*p* < 0.001, d = 6.74).

*Adiponectin* also has anti-inflammatory properties. It is mainly secreted by adipose tissue, but also produced in other tissues, such as skeletal muscle [48]. In skeletal muscle specifically, *Adiponectin* has been shown to increase oxidative capacity [49] and to promote muscle function, development, maintenance and growth [26]. Our data show that *Adiponectin* mRNA expression decreased in adult and old mice compared with young mice (*p* = 0.002, d = −2.02 and *p* < 0.001, d = −2.95, respectively) (Figure 1A).

With regard to muscle damage, we analyzed markers of atrophy and markers of apoptosis. One of the various pathways regulating muscle protein breakdown is the ubiquitin proteasome pathway, which ultimately activates the muscle-specific E3 ubiquitin ligases (Atrogin-1 and Murf1), which role is to target protein for proteolysis [50]. Therefore, they are considered markers of muscle atrophy [51]. Both atrophy markers *Atrogin-1* and *Murf1* showed a noticeable decrease in mRNA expression in old mice compared with young mice (*p* < 0.001, d = −3.21 and *p* < 0.001, d = −5.51, respectively). The decrease was already statistically significant in adult mice for *Murf1* (*p* = 0.003, d = −1.85) (Figure 1B).

Caspases are a family of genes involved in apoptosis and inflammation [52]. We analyzed the expression of *Caspase-8* and *Caspase-9* (initiators of apoptosis) and of *Caspase-3* (executioner of apoptosis). The mRNA expression of *Caspase-8* increased significantly in adult mice (*p* = 0.009, d = 1.66). At 18 months, the expression of *Caspase-3* and *-8* was significantly lower compared with adult mice (*p* = 0.002, d = −1.98 and *p* < 0.001, d = −2.32, respectively), while *Caspase-9* mRNA expression was significantly reduced compared with young mice (*p* = 0.045, d = −1.21).

Muscle regeneration genes were also studied. We selected *Myod*, *Myog* and *Myf5*, which are genes involved in myogenesis regulation, muscle cell differentiation and muscle regeneration [53]. As Figure 1C shows, no changes were detected in *Myf5* mRNA expression. On the other hand, the expressions of *Myod* and *Myog* decreased in adult mice compared with young ones (*p* = 0.049, d = −1.51 and *p* < 0.001, d = −8.47, respectively).

We also compared the expression of these muscle genes between 18-month-old lean and DIO mice. *Tnf-α*, *Adiponectin*, *Caspase-3* and *Myod* mRNA expression was significantly higher in the old DIO group compared with old lean mice (*p* < 0.001, *p* < 0.001, *p* = 0.020 and *p* = 0.002, respectively), while the expression of *Myf5* and *Myog* was lower (*p* = 0.049 and *p* < 0.001) in the DIO group compared to the 18-month-old lean mice. There were no other significant differences between old and DIO mice in the other genes analyzed related to inflammation, muscle damage and muscle regeneration (Figure S2A–C).

#### 3.1.3. Effects on Genes and Proteins Related to Muscle Metabolism

**Figure 2:**
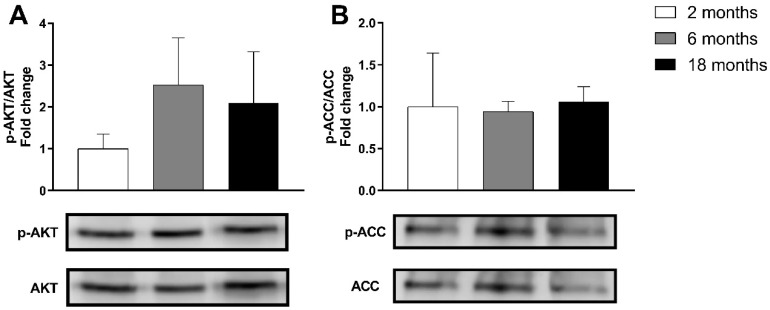
Representative Western blot and densitometry analysis of the effects of aging (2, 6 and 18 months) on phosphorylated AKT and total AKT (**A**) and phosphorylated ACC and total ACC (**B**) in the gastrocnemius muscle of C57BL/6J female mice fed a standard diet. Band densities of phosphorylated AKT and ACC were normalized by total AKT and ACC, respectively. Data presented as mean (SD); *n* = 3–4.

GLUTs are a family of transmembrane proteins involved in the transport of glucose across cellular membranes. As shown in Figure 1D, the mRNA expression of glucose transporters *Glut1* and *Glut4* decreased significantly in adult mice (*p* < 0.001, d = −2.33 and *p* = 0.01, d = −7.07, respectively) and in old mice (*p* < 0.001, d = −5.63 and *p* < 0.001, d = −7.60, respectively), compared with young mice. Skeletal muscle insulin signaling is a major determinant of muscle growth and glucose homeostasis. AKT is a Serine/Threonine protein kinase activated by the phosphorylation of Ser473, that plays a key role in mediating insulin signaling and glucose uptake [54]. The analysis of the p-AKT/AKT ratio revealed no significant changes induced by aging in the gastrocnemius muscle (Figure 2).

The beta-oxidation pathway is the main source of fatty acid oxidation (FAO), which mainly occurs in the mitochondria [55]. Since long-chain FAs cannot cross the mitochondrial membrane through simple diffusion, they must be converted to acetyl-CoA and then transported into the mitochondrial matrix by the CPT system, CPT1b being the isoform found in skeletal muscle [56]. On the other hand, very long FAs are oxidized in peroxisomes, the ACOX enzyme being the first one in the peroxisomal beta-oxidation pathway [57]. A decrease in the mRNA expression of *Cpt1b* was observed in adult mice in comparison with young ones (*p* = 0.022, d = −1.51). However, its expression experimented a significant increase in old mice compared with young ones (*p* = 0.004, d = 1.74). *Acox* experimented similar changes: a decrease was observed in adult mice (*p* = 0.024, d = −1.80), which was reversed in old mice (*p* < 0.001, d = 1.96) (Figure 1D). One of the major controllers of FAO is Acetyl-CoA Carboxylase (ACC), which converts Acetyl-CoA to malonyl-CoA, which is the first step in the synthesis of FAs, but also acts as a CPT1 inhibitor. ACC is inactivated by phosphorylation [58]. As shown in Figure 2B, no statistically significant changes were detected due to aging in p-ACC/ACC ratio.

In the comparison between old lean and old DIO mice, the expression of *Glut4* mRNA was significantly higher in the DIO group (*p* < 0.001), while the p-AKT/AKT ratio was significantly lower (*p* = 0.025) in that group. No other significant differences were found in other genes and proteins related to glucose uptake and FAO (Figure S2D,F).

#### 3.1.4. Effects on mRNA Expression of Genes Related to Myokines

The expression of genes encoding muscle myokines is presented in Figure 1E. The FNDC5 gene encodes the adipomyokine Irisin [59]. Its main roles include the browning of WAT and the activation of genes related to thermogenesis [15]. Furthermore, plasma Irisin levels have been shown to predict telomere length in humans [60]. Our data show that the mRNA expression of *Fndc5* experimented a significant increase in adult mice (*p* = 0.024, d = 1.40), but that increase was reversed in old mice (*p* < 0.001, d = −2.50).

Meteorin-like (Metrnl) promotes the expression of anti-inflammatory cytokines, is involved in the adaptations to cold exposure and WAT browning [16], acts as a neurotrophic factor [61] and has insulin sensitizing effects [62,63]. *Metrnl* mRNA expression was almost completely suppressed in old mice (*p* < 0.001, d = −7.43).

Interleukin-6 (IL-6) is a ubiquitous cytokine, which effects could vary depending on where and why it is secreted [64]. Figure 1E shows that *Il-6* mRNA expression was significantly reduced in the muscle of adult and old mice compared to young mice (*p* < 0.001, d = −2.77 and *p* = 0.003, d = −1.72, respectively).

Myostatin, also known as growth differentiation factor-8, is a myokine part of the transforming growth factor-β family that serves as a negative regulator of skeletal muscle growth [65,66]. In the present study, no significant changes were observed in the mRNA expression of *Myostatin* due to aging.

Concerning the differences between 18-month-old lean and DIO mice, a significant upregulation of *Fndc5* (*p* < 0.001) and *Metrnl* (*p* < 0.001) mRNA expression was found in the DIO group, with no significant changes in *Il-6* or *Myostatin* (Figure S2E).

### 3.2. Effects of Long-Term DHA Supplementation and/or Treadmill Training on Muscle in DIO Old Mice

#### 3.2.1. Effects on Whole Body Lean Mass and Muscle Relative Weights

As described in previous studies of our group, no significant changes were observed in BW or fat mass after long-term DHA supplementation and/or physical exercise [39,40]. In addition, no significant differences were observed between groups regarding absolute and relative lean mass. Furthermore, gastrocnemius and soleus absolute weights (data not shown) and relative mass were not modified either by DHA or exercise (Table 3).

#### 3.2.2. Effects on mRNA Expression of Pro-Inflammatory, Muscle Damage- and Muscle Regeneration-Related Genes

**Figure 3:**
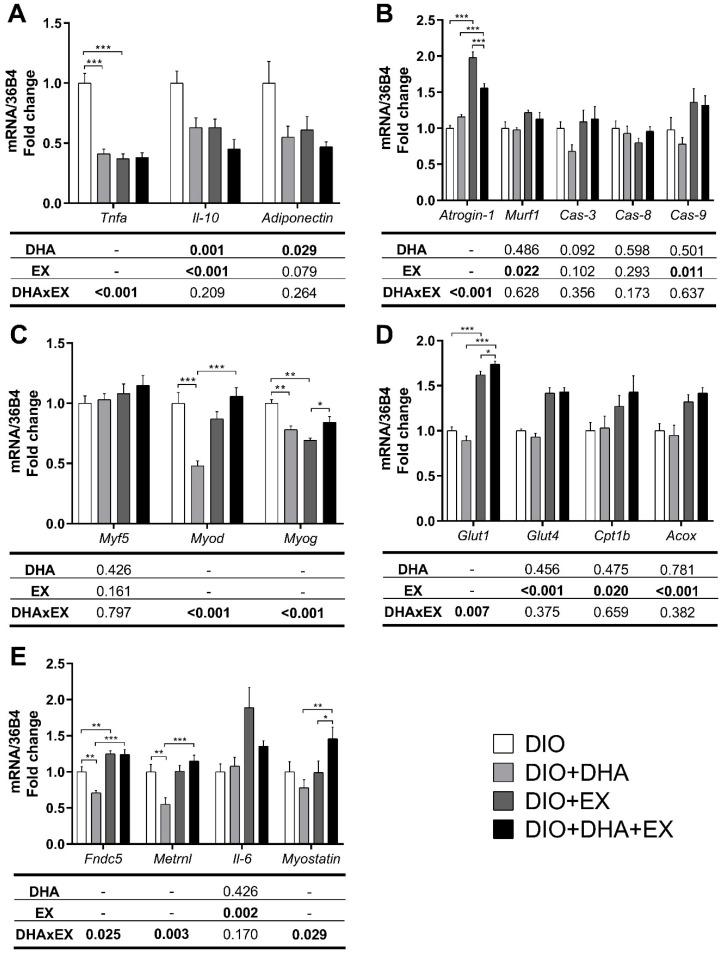
Effects of long-term DHA supplementation and/or exercise training on genes related to inflammation (**A**), muscle damage (**B**), muscle regeneration (**C**), glucose uptake and fatty acid oxidation (**D**) and myokine expression (**E**) in the gastrocnemius muscle of 18-month-old DIO female mice. Data presented as mean (SD); *n* = 6–10. DIO: Diet-induced obese; EX: Treadmill training. For two-way ANOVA, when an interaction was found, contrast analysis was performed. The *p* values for the main factors, DHA supplementation (DHA), exercise (EX) and the interaction between both (DHAxEX) are shown in the corresponding rows of the tables below the figures. Statistically significant *p* values are marked in bold. Significant differences between groups are shown above the corresponding comparisons: * *p* < 0.05, ** *p* < 0.01, *** *p* < 0.001.

An interaction was detected between DHA and treadmill training in *Tnf-α* mRNA expression (*p* < 0.001). The contrast analysis showed significant decreases in the exercise and in the DHA group compared to the DIO group (both *p* < 0.001, d = −4.01 and d = −3.79, respectively). The two-way ANOVA also revealed that the treatments decreased *Il-10* mRNA expression (*p* = 0.001 for DHA-supplemented vs. non-supplemented groups; *p* < 0.001 for exercise-trained vs. non-trained groups), while only DHA reduced the mRNA expression of *Adiponectin* (*p* = 0.029) (Figure 3A).

With regard to *Atrogin-1*, another interaction between DHA and exercise was observed (*p* < 0.001). The contrast analysis revealed a significant increase (*p* < 0.001, d = 5.88) in *Atrogin-1* mRNA expression in the exercise group compared to the DIO group, with no changes in the group receiving DHA alone. Interestingly, the increase in *Atrogin-1* expression caused by exercise was ameliorated in the presence of DHA (*p* < 0.001, d = 2.46). Moreover, exercise induced significant increases in *Murf1* and *Caspase-9* mRNA expression, independently of DHA (*p* = 0.022 and *p* = 0.011, respectively). No significant changes were observed on *Caspase-3* and *-8* due to the treatments (Figure 3B).

Figure 3C shows the expression of genes related to muscle regeneration. No changes were detected in *Myf5* expression due to the treatments, but significant interactions (*p* < 0.001) between DHA and exercise were found for *Myod* and *Myog*. Compared to the DIO group, *Myod* expression was significantly reduced in the DHA group (*p* < 0.001, d = −3.02). However, that decreased expression was reversed in the group that combined DHA and exercise, compared to the DHA alone group (*p* < 0.001, d = 3.30). As for *Myog*, reduced expressions were found in the DHA group (*p* = 0.003, d = −2.50) and in the exercise group (*p* = 0.003, d = −3.64), compared to the DIO group, while in the presence of exercise, DHA increased *Myog* expression compared to exercise alone (*p* = 0.010, d = 1.85).

#### 3.2.3. Effects on Genes and Proteins Related to Muscle Metabolism

**Figure 4:**
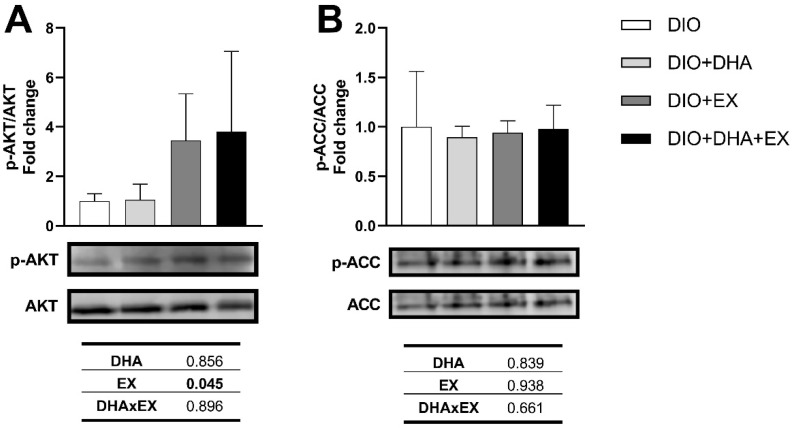
Representative Western blot and densitometry analysis of the effects of long-term DHA supplementation and/or exercise training on phosphorylated AKT and total AKT (**A**) and phosphorylated ACC and total ACC (**B**) in the gastrocnemius muscle of 18-month-old DIO female mice. Band densities of phosphorylated AKT and phosphorylated ACC were normalized by total AKT and total ACC, respectively. Data presented as mean (SD); *n* = 3–4. The *p* values for the main factors, DHA supplementation (DHA), exercise (EX) and the interaction between both (DHAxEX) are shown in the corresponding rows of the tables below the figures. Statistically significant *p* values are marked in bold.

The two-way ANOVA revealed a significant interaction between DHA and exercise in *Glut1* mRNA expression (*p* = 0.007), with the contrast analysis showing a significant increase in the exercise group compared with the DIO group (*p* < 0.001, d = 5.59). In the presence of DHA, exercise further increased *Glut1* expression compared to exercise alone (*p* = 0.029, d = 1.12). *Glut4* mRNA expression increased in the trained groups vs. the non-trained groups (*p* < 0.001) (Figure 3D). In parallel, a significant increase was found in p-AKT/AKT ratio in the exercise groups (*p* = 0.045, 3.5-fold and 3.8-fold for DIO + EX and DIO + DHA + EX, respectively) compared with the DIO group (Figure 4A). Exercise also increased the mRNA expressions of *Cpt1b* (*p* = 0.020) and *Acox* (*p* < 0.001), while DHA supplementation alone did not cause any significant changes in the expression of genes involved in fatty acid oxidation (Figure 3D). On the other hand, no significant changes were detected in p-ACC/ACC ratio by any of the treatments (Figure 4B).

#### 3.2.4. Effects on mRNA Expression of Genes Related to Myokines

Figure 3E shows an interaction between treatments in *Fndc5* mRNA expression (*p* = 0.025). The contrast analysis revealed that its expression increased in the exercise group in comparison with the DIO group (*p* = 0.003, d = 1.50), while DHA alone significantly reduced it (*p* = 0.002, d = −1.75). *Fndc5* expression increased in the group combining DHA and exercise, compared with DHA alone (*p* < 0.001, d = 3.17). In the case of *Metrnl*, an interaction was also found (*p* = 0.003). As observed with the expression of *Fndc5*, *Metrnl* mRNA expression decreased in the DHA group (*p* = 0.002, d = −1.76) and it increased again in the group combining exercise and DHA, compared to DHA alone (*p* < 0.001, d = 2.34). Moreover, exercise exerted a significant increase in muscle *Il-6* mRNA expression regardless of DHA supplementation (*p* = 0.002). Lastly, another interaction between treatments was detected for *Myostatin* (*p* = 0.029). The contrast analysis revealed a significant increase in *Myostatin* mRNA expression in the DHA + EX group compared to the DHA group (*p* = 0.004, d = 1.61) and compared to the exercise alone group (*p* = 0.028, d = 1.12).

## 4. Discussion

Aging is typically associated with an altered regulation of the inflammatory response, characterized by increased levels of inflammatory cytokines and an impaired glucose metabolism [7,67]. In agreement with previous studies, our current data show an increase in the mRNA expression of the pro-inflammatory *Tnf-α* in old (18-month-old) mice compared with young (2-month-old) mice [68,69]. Likewise, increases in *Tnf-α* mRNA expression in obese mice have been widely reported in the literature [70,71]. In our study, the aged obese mice had the highest expression of this cytokine, suggesting an increased muscle inflammatory status when aging and obesity concurred. In parallel, a decreased muscle mRNA expression of *Adiponectin* was found over time. *Adiponectin* has anti-inflammatory and insulin-sensitizing properties [72] and TNF-α has been shown to inversely modulate *Adiponectin* expression [73], further supporting the increased inflammation in the skeletal muscle of aged mice. However, its expression increased in the aged obese mice, contrary to what is generally observed in obesity [26]. Surprisingly, the expression of the anti-inflammatory *Il-10* increased with aging. This increase has already been described in skeletal muscle [68] and in a previous study of our group conducted in BAT [40].

We also observed marked changes in genes related to glucose uptake and FAO. A progressive downregulation in the mRNA expression of *Glut1* and *Glut4* was observed with aging: the expression of both genes decreased in adult mice, with a further decline in *Glut1* expression in old mice. A study of our group that analyzed the same tissues found no significant changes in GLUT4 protein expression with aging [45]; however, previous studies have found progressive declines in GLUT4 protein and *Glut4* mRNA expression [74,75,76]. Unexpectedly, the mRNA expression of *Glut4* increased in the aged and obese mice, while decreases or no changes are usually reported [77,78]. Then, we studied p-AKT/AKT protein expression, but no significant changes were found due to aging, while previous rodent studies have reported unclouding results [69,79,80,81]. Importantly, in agreement with previous literature [82,83], a significant decrease in the p-AKT/AKT ratio was found in the aged obese mice. On the other hand, the expression of *Cpt1b* increased significantly in old mice compared with young mice, while no changes were found in neither *Acox* mRNA expression nor in p-ACC/ACC ratio. In the literature, decreases in p-ACC due to aging [76,84], as well as no changes [80], have been reported. In this sense, the changes in mRNA expression might suggest a switch from a glycolytic to a more oxidative metabolism in order to sustain skeletal muscle energy supply, which might reflect a potential switch in fiber type, with decreases in the glycolytic type II fibers and the resulting relative increases in the more oxidative type I fibers [85]. However, we have to be cautious with this hypothesis, as no significant changes were observed at the protein level, and therefore further research is needed in order to elucidate this hypothesis, although it could be further supported by the increase in *Tnf-α* mRNA expression that we observed with aging, since TNF-α is linked to IR [86,87]. Lastly, aging coupled with obesity seemed to further increase muscle inflammatory status and worsen glucose metabolism, as supported by the increase in *Tnf-α* mRNA expression and the decreased p-AKT/AKT ratio found in the aged obese mice.

In relation to myokine production, an initial increase in *Fndc5* mRNA expression was found in adult mice, but at 18 months the expression was similar to that of young mice. However, obesity increased *Fndc5* expression compared with the aged lean mice. Studies show conflicting results [88,89,90] and, to our knowledge, no studies to date have analyzed muscle *Fndc5* expression in aging mice. Considering Irisin’s ability to predict telomere length (a reliable marker of aging) [60] and Irisin overall associations with positive health outcomes [91], the observed results were unforeseen. Likewise, changes in *Metrnl* mRNA expression in skeletal muscle due to aging have not been reported, to our knowledge. We found a decreased *Metrnl* mRNA expression in adult mice (50%) and it was almost suppressed in old mice. Similarly, significant decreases were found in *Il-6* mRNA expression in adult and in old mice compared with young ones. Taking into account that IL-6 has been proposed as an energy sensor within muscles (especially for muscle glucose uptake) [64], that *Metrnl* promotes an anti-inflammatory environment, improves glucose metabolism and reduces insulin resistance (IR) [62,63]; and considering that in our mice aging decreased the mRNA expressions of *Glut1* and *Glut4* and increased the expression of *Tnf-α*, the decreases observed in the expression of these myokines seems coherent. Lastly, glucose serum levels in the old mice were significantly higher in the old mice than in the young, as reported in a previous study of our group [40], supporting the idea of an unhealthier glucose metabolism control. With regards to *Myostatin* mRNA expression, no significant changes were observed due to aging, while previous rodent studies have shown mixed results [92,93].

Aging is also associated with the loss of muscle mass. In our study, the relative lean mass was significantly lower in old mice compared with adult and young ones. Moreover, we observed a marked decrease in the mRNA expression of the muscle-specific E3 ubiquitin ligases (*Atrogin-1* and *Murf1*) in old mice, in agreement with previous studies [51,94]. Nevertheless, increases in the expression of *Atrogin-1* and *Murf1* have also been reported [69]. The discrepancies may be explained by the use of different animal models and muscles. Importantly, it has been suggested that accurately measuring the expression of these genes over time is difficult [95]. However, muscle atrophy can be caused by other factors such as apoptosis, which is activated by Caspases. We found a significant decrease in the mRNA expression of *Caspase-9* in old mice compared with young ones, in agreement with a previous study in rats that also analyzed the gastrocnemius muscle [96]. Obesity did not cause changes in markers of muscle damage, with the sole exception of an increase in the mRNA expression of *Caspase-3* compared with the lean aged mice. In view of the decreased expression of atrophy markers in old mice (which had approximately 25% less relative lean mass), markers of muscle regeneration were studied. We only found decreased mRNA expressions of the myogenic factors *Myod* and *Myog* in adult mice. A similar pattern in the expression of these genes due to aging has been reported previously [97]. However, *Myf5* expression did not change in our mice with aging, as observed in a previous study in mice [98]. Obesity in the aged mice seemed to alter this pattern, with a decreased mRNA expression of *Myf5* and *Myog*, and increased *Myod* expression compared with the aged lean mice. These findings could imply a reduced protein turnover in the skeletal muscle of aged mice, but more research is needed.

Exercise, on the other hand, opposes most of the effects associated with aging and obesity. For instance, exercise has been shown to reduce *Tnf-α* overexpression in mice [99]. Indeed, we found a decreased *Tnf-α* expression in the exercise group. Similarly, DHA alone was also able to reduce *Tnf-α* expression. This has been reported in previous studies [100,101,102,103]. Concerning *Il-10*, the decreased mRNA expression induced by both interventions could imply a lower need for *Il-10* anti-inflammatory properties. This is supported by the reduced inflammatory state expected in mice performing exercise [104] and by DHA anti-inflammatory properties observed in skeletal muscle [105]. Furthermore, compensatory increases in *Il-10* levels have been reported as a consequence of acute exercise bouts [106], which increase inflammation, contrary to chronic exercise. Taking everything into account, the decreases in *Il-10* expression observed in mice receiving DHA and/or performing chronic exercise, as well as the increases previously observed with aging, seem reasonable. For *Adiponectin*, a lower mRNA expression was observed in the DHA groups, while exercise did not cause significant changes. DHA has been reported to increase *Adiponectin* mRNA expression and *Adiponectin* levels [107,108,109]. Thus, our findings might be partially explained by the “*Adiponectin* paradox” [110], in which *Adiponectin* is elevated in aged people with obesity and IR. In this context, reductions in *Adiponectin* expression could indicate a positive result. In fact, in our study *Adiponectin* mRNA expression was significantly higher in the obese aged group than in the aged lean mice. Moreover, muscle *Adiponectin* expression has been positively associated with intramuscular triglycerides (IMTG) [48] and IMTG reductions caused by DHA have been reported [105,111], which might also explain the reduced *Adiponectin* expression observed.

In addition, exercise increases energy demands and glucose uptake in skeletal muscle [112]. We found increases in the mRNA expressions of *Glut1* and *Glut4* in the exercise groups, while DHA alone did not have an effect. Similar increases have been reported in rodents and myotubes [113,114,115,116]. It is also well established that FAO increases with exercise, as energy demands increase compared to rest [117,118,119]. We observed an upregulated expression of *Cpt1b* and *Acox* (genes related to mitochondrial and peroxisomal FAO, respectively) in all exercise-performing mice. Similarly, previous studies have shown that exercise increases ACOX1 and CPT1 levels in adipose tissue [120] and CPT1 protein expression in skeletal muscle [121]. Our protein analysis did not detect significant changes in p-ACC/ACC ratio, in agreement with previous studies in aged rats [79], but we did find an almost four-fold increase in p-AKT/AKT ratio in the exercise-performing mice, as supported by previous studies [122,123,124]. Although we have not measured the expression of GLUT4 in the plasma membrane, the increased phosphorylation of AKT suggests an activation of the PI3K/AKT pathway, which is involved in the insulin-stimulated translocation of GLUT4 from cytoplasmatic vesicles to the plasma membrane [54]. Altogether, our findings suggest that exercise was able to improve muscle glucose metabolism and insulin sensitivity in old and obese mice.

Besides, several myokines are secreted during exercise. Exercise is associated with changes in Irisin levels [89], but the extensive literature on Irisin and exercise shows some contradictory results [15,125]. We found a significant increase in the expression of *Fndc5* in the exercise group, coherent with part of the literature, while DHA alone seemed to reduce its expression. Interestingly, exercise in combination with DHA was able to reverse the decreased expression observed in the DHA alone group. Exercise also induces *Metrnl* expression in skeletal muscle [16] and studies in exercise-performing mice have found increases in muscle Metrnl protein, serum levels and/or *Metrnl* mRNA [16,120,126,127]. However, we found no significant changes in *Metrnl* expression due to exercise in comparison with the DIO mice. Nevertheless, it is relevant to consider that our study is carried out in aged obese mice, which could have affected the muscle capability to produce *Metrnl* in response to exercise. Notably, in contrast to all the commented studies, we used females, which could also account for a differential response. As previously observed with *Fndc5*, DHA alone significantly decreased *Metrnl* mRNA expression compared with the other groups, but when it was combined with exercise the decrease was reversed. It has been widely demonstrated that exercise can upregulate *Il-6* mRNA expression in active skeletal muscle [128,129,130], in agreement with our results.

Lastly, we observed several changes caused by the treatments in the expression of genes related to muscle damage and muscle regeneration. Exercise increased the mRNA expressions of *Atrogin-1* and *Murf1*. Similar results have been observed in rats [131]. Exercise has also been described to upregulate apoptotic pathways within the muscle [132,133]. Indeed, we observed that exercise significantly increased the mRNA expression of *Caspase-9*. Equivalent results have been previously reported [134,135]. Since exercise-performing mice had an increased expression of some atrophy markers while having the same lean mass as sedentary mice, a compensatory increase in the expression of myogenic factors (*Myf5*, *Myod* and *Myog*) could be expected [136]. Nevertheless, we did not observe a compensatory increase. In fact, *Myog* mRNA expression decreased in the exercise group compared with the DIO mice, while the expressions of *Myod* and *Myf5* were not affected by exercise. Considering that no differences in total and relative lean mass were found between treated groups, the increases observed in atrophy markers could suggest that exercise might increase the rate of muscle protein turnover. When looking at DHA alone on markers of muscle damage, no significant changes were found, but DHA alone decreased the mRNA expression of the myogenic factors *Myod* and *Myog*. Moreover, several interactions were found between DHA and exercise: *Atrogin-1* expression increased in the exercise groups, but the increase was significantly ameliorated in the group that also included DHA. Similar outcomes have been observed in myotubes [137]. Likewise, the decreased expression of *Myod* observed in the DHA group was reversed in the DHA and exercise group. These data highlight the complexity of the regulation of genes related to muscle damage and muscle regeneration during aging and obesity in response to exercise or dietary composition.

Regarding the changes observed in the mice supplemented with DHA, it should be also considered that the effects found might have also been related to the consequent decrease in the ratio of omega-6 to omega-3 fatty acids (*n*-6/*n*-3 ratio) and not only caused by the increase in DHA per se. Several studies have shown the relevance of the *n*-6/*n*-3 ratio in the muscle function and in the risk to develop obesity and related metabolic disorders [138,139].

### Strengths and Limitations

The main strength of the present study is the use of long-term treatments. Both DHA supplementation and aerobic exercise were followed for 12 months, while most of the previous studies involved shorter periods. Other strengths are the analysis of the effects of the treatments alone or in combination with each other and the use of female mice. Most of the previous studies to date regarding sarcopenia and aging, as well as *n*-3 PUFA supplementation and exercise, have been carried out in male mice [35,36,37], which was one of the reasons for choosing females in this study. However, it is important to consider that some studies have reported metabolic differences between male and female mice during aging and obesity [140] and therefore the use of only female mice could also be seen as a limitation, since results could vary between sexes. In our study, we did not evaluate the estrous cycles or estrogen levels, although several studies have shown that 18-month-old C57BL/6J female mice are acyclic [141]. Another limitation of the present study is the fact that the differences between groups were analyzed mainly at the gene expression level. Further studies more focused at the protein/enzymatic level, as well as on the characterization of contractile fibers and oxidative stress, would be of interest in order to reach more definitive conclusions about the complex interactions of aging and obesity, as well as the effects of the treatments with DHA and/or exercise on muscle metabolism and function. It would have been also relevant to determine if the changes observed at organ/tissue level would have been reflected on whole body energy expenditure and activity; however, technical problems with the equipment did not allow us to measure these parameters. Finally, although we do not provide here any direct evidence of improved fitness, it is important to mention that we have shown in previous studies that exercise alone or in combination with DHA has relevant effects on improving markers of hepatic steatosis and liver inflammation [39].

## 5. Conclusions

Our results suggest that aging alters the expression of genes involved in muscle inflammation, glucose uptake and protein degradation and regeneration, as well as the expression of some myokines in mice following a control diet. Some of these changes were aggravated in aged obese mice. In these mice, long-term exercise training had more relevant effects on muscle than DHA. Indeed, exercise (independently of DHA supplementation) counteracted some of the alterations induced by aging and obesity on genes encoding inflammatory markers, glucose transporters and fatty acid oxidation regulators, in parallel with an improvement in insulin signaling and a partial restoration of the altered myokine expression. On the other hand, DHA regulated the expression of some genes related to inflammation, myokine expression and muscle regeneration. The current data should be considered in the context of our previously reported effects for DHA and/or exercise in aged obese female [39,40,142], suggesting a contribution of the hepatic-adipose-muscle axis in mediating the beneficial effects of these treatments on lipid and glucose metabolism and on hepatic steatosis.

## Figures and Tables

**Table 1:** Mouse primers sequences for SYBR GREEN RT-PCR.

Gene	Forward Primer Sequence	Reverse Primer Sequence
*Acox*	CTATGGGATCAGCCAGAAAG	AGTCAAAGGCATCCACCAA
*Adiponectin*	CGAGGATTCTCTGGAACTGC	GGTCGCTTCTTCAAGGTCTG
*Atrogin-1*	CTTTCAACAGACTGGACTTCTCGA	CAGCTCCAACAGCCTTACTACGT
*Caspase-3*	CCTCAGAGAGACATTCATGG	GCAGTAGTCGCCTCTGAAGA
*Caspase-8*	ACCGAGATCCTGTGAATGGAACC	TAAGAATGTCATCTCCTTGAGGA
*Caspase-9*	AGTTCCCGGGTGCTGTCTAT	GCCATGGTCTTTCTGCTCAC
*Cpt1b*	CGAGGATTCTCTGGAACTGC	GGTCGCTTCTTCAAGGTCTG
*Fndc5*	GGTGCTGATCATTGTTGTGG	CGCTCTTGGTTTTCTCCTTG
*Glut1*	TCAACACGGCCTTCACTG	CACGATGCTCAGATAGGAC
*Glut4*	AAAAGTGCCTGAAACCAGAG	TCACCTCCTGCTCTAAAAGG
*Il-6*	GAGGATACCACTCCCAACAGACC	AAGTGCATCATCGTTGTTCATACA
*Il-10*	AAGGCAGTGGAGCAGGTGAA	CCAGCAGACTCAATACACAC
*Metrnl*	AAGCCTTTCAGGGACTCCTC	CCCTGGTCGTACTCCACACT
*Myf5*	CACCAACCCTAACCAGAGACTCCC	GCTGTTACATTCAGGCATGCCG
*Myod*	CGCGCTCCAACTGCTCTGATGG	CTCGACACAGCCGCACTCTTCC
*Myog*	GACCCTACAGACGCCCACAATC	ACACCCAGCCTGACAGACAATC
*Myostatin*	TGCAAAATTGGCTCAAACAG	GCAGTCAAGCCCAAAGTCTC
*Murf1*	GCTGGTGGAAAACATCATTGACAT	CATCGGGTGGCTGCCTTT
*Tnf-α*	CATCTTCTCAAAATTCGAGTGACAA	TGGGAGTAGACAAGGTACAACCC
*36b4*	CACTGGTCTAGGACCCGAGAAG	GGTGCCTCTGGAGATTTTCG

Primers were specific for mouse (Mus musculus). *Acox*: acetyl-CoA Oxidase 1, *Atrogin-1*: muscle atrophy F-Box, *Cpt1b*: carnitine palmitoyl-transferase 1B, *Fndc5*: fibronectin type III domain containing 5, *Glut1*: glucose transporter 1, *Glut4*: glucose transporter 4, *Il-6*: interleukin 6, *Il-10*: interleukin 10, *Metrnl*: meteorin-like, *Myf5*: myogenic factor 5, *Myod*: myogenic differentiation 1, *Myog*: myogenin, *Murf1* muscle-specific RING finger protein 1, *Tnf-α*: tumor necrosis factor alpha, *36b4*: ribosomal protein lateral stalk subunit p0.

**Table 2:** Whole-body lean mass and gastrocnemius and soleus relative weights in young (2 months), adult (6 months) and old (18 months) mice.

Parameter	2 Months (*n* = 10)	6 Months (*n* = 7)	18 Months (*n* = 9)
Lean mass (g)	13.85 (0.55)	15.96 (0.50) ***	16.74 (0.74) *** ^#^
Lean mass (%)	77.84 (2.63)	76.49 (2.74)	57.76 (9.78) *** ^###^
Gastrocnemius (%BW)	0.50 (0.06)	0.47 (0.06)	0.44 (0.05)
Soleus (%BW)	0.04 (0.02)	0.03 (0.01) *	0.03 (0.00) **

Data presented as mean (SD). BW: Body weight. * *p* < 0.05, ** *p* < 0.01, *** *p* < 0.001 vs. 2-month-old mice; ^#^ *p* < 0.05, ^###^ *p* < 0.001 vs. 6-month-old mice.

**Table 3:** Effects of long-term DHA supplementation and/or exercise on whole body lean mass and on gastrocnemius and soleus muscle relative mass in old DIO mice.

Parameter	DIO (*n* = 10)	DIO + DHA (*n* = 6)	DIO + EX (*n* = 8)	DIO + DHA + EX (*n* = 9)	2 × 2 ANOVA		
					DHA	EX	DHAxEX
					*p*	*p*	*p*
Lean mass (g)	20.30 (1.66)	18.85 (0.67)	19.84 (1.68)	20.27 (0.95)	0.293	0.324	0.052
Lean mass (%)	38.27 (4.44)	37.48 (2.86)	37.18 (1.65)	39.05 (3.61)	0.647	0.834	0.265
Gas (%BW)	0.25 (0.04)	0.27 (0.05)	0.27 (0.04)	0.26 (0.04)	0.687	0.687	0.232
Soleus (%BW)	0.02 (0.00)	0.02 (0.00)	0.02 (0.00)	0.02 (0.00)	0.971	0.754	0.71

Data presented as mean (SD). BW: Body weight, DIO: Diet-induced obese, EX: Treadmill training, Gas: Gastrocnemius.

## Data Availability

Not applicable.

## Supplementary Materials

The following supporting information can be downloaded at: https://www.mdpi.com/article/10.3390/nu14204240/s1, Figure S1: Effects of a long-term obesogenic diet on: whole-body lean mass, relative lean mass and gastrocnemius and soleus muscle relative mass in aged (18-month-old) C57BL/6J female mice. Figure S2: Effects of a long-term obesogenic diet on the mRNA expression of genes related to inflammation, muscle damage, muscle regeneration, glucose uptake and fatty acid oxidation and myokine expression and on phosphorylated AKT/total AKT and phosphorylated ACC/total ACC ratios, in the gastrocnemius muscle of aged (18-month-old) C57BL/6J female mice.
