# Supplementary material for: Changes Induced by Aging and Long-Term Exercise and/or DHA Supplementation in Muscle of Obese Female Mice
Source: Nutrients. 2022 Oct 12;14(20):4240. doi: 10.3390/nu14204240 (PMC9610919; doi:10.3390/nu14204240)
Supplement: Supplementary file 1 [file nutrients-14-04240-s001.zip › nutrients-1855183-supplementary.pdf]

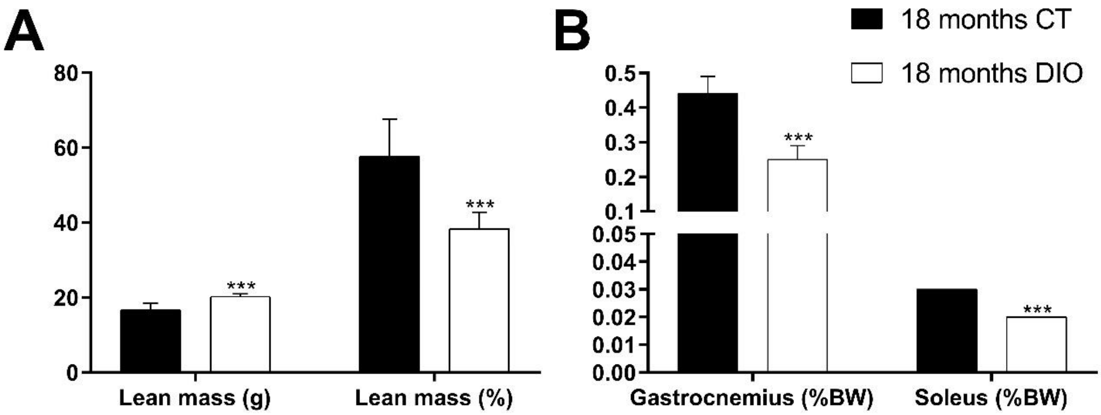

Figure S1. Effects of a long-term obesogenic diet on whole-body lean mass (**A**), and on gastrocnemius and soleus muscle relative mass (**B**), in aged (18-month-old) C57BL/6J female mice. Data presented as mean (SD); *n* = 9-10. *** $p < 0.001$ vs. 18 months control (CT).

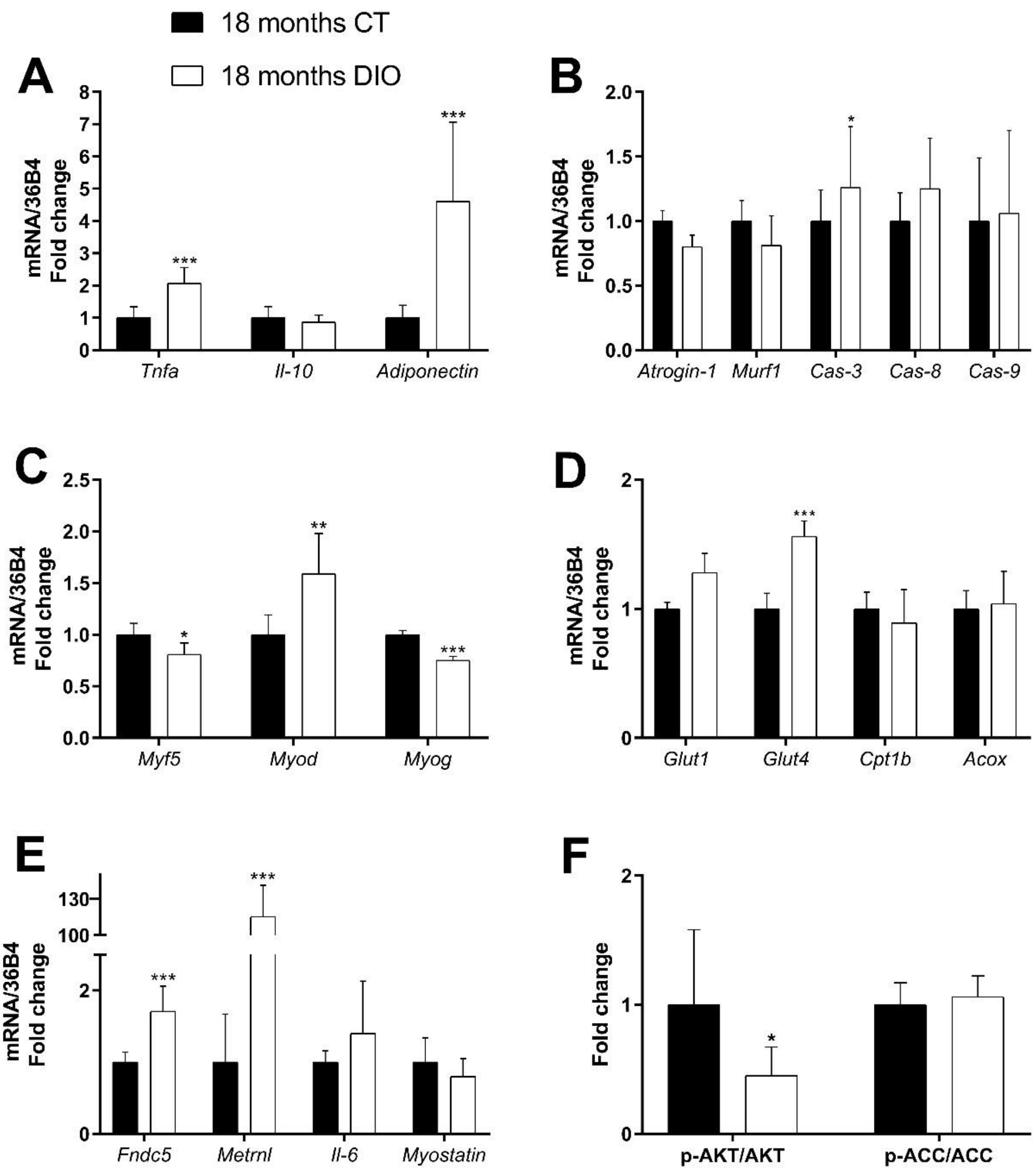

Figure S2. Effects of a long-term obesogenic diet on the mRNA expression of genes related to inflammation (**A**), muscle damage (**B**), muscle regeneration (**C**), glucose uptake and fatty acid oxidation (**D**), and myokine expression (**E**), and on phosphorylated AKT/total AKT and phosphorylated ACC/ total ACC ratios (**F**), in the gastrocnemius muscle of aged (18-month-old) C57BL/6J female mice. Data presented as mean (SD); n = 3-10. * $p < 0.05$, ** $p < 0.01$, *** $p < 0.001$ vs. 18 months control diet (CT).
